# Supplementary material for: The effect of conflict on damage to medical facilities in Mariupol, Ukraine: A quasi-experimental study
Source: PLOS Glob Public Health. 2025 Jan 15;5(1):e0003950. doi: 10.1371/journal.pgph.0003950 (PMC11734947; doi:10.1371/journal.pgph.0003950)
Supplement: Attachment — Submitted filename: Response_to_reviewers_2024-08-02.docx [file pgph.0003950.s002.docx]

07 August 2024

Dear Editorial Office,

**RE: PGPH-D-24-00764 - “The effect of conflict on medical facilities in Mariupol, Ukraine: a quasi-experimental study”**

We thank the Editor and Reviewers for their thoughtful and important comments. We have carefully addressed each comment in a revised version of our manuscript. Below, we describe the point-by-point changes in response to the Editor’s and Reviewers’ comments. The line numbers in our responses refer to those in the tracked-change version of the updated manuscript). We believe that the significant revisions in response to the Editor and Reviewer points have greatly improved the manuscript.

**Editor report:**

**Thank you for sharing your important analysis.

Please pay particular attention to the comments by reviewer 2 on:
- The risk of misattribution of ACCLED data and improving its alignment with building damage. Do you need this analysis, given the lack of temporal alignment?**

Thank you for raising this issue. We have reconsidered including the ACLED data in our analysis due to the lack of temporal alignment. In reading the Reviewer reports, it is clear that the weaknesses associated with the linkage of ACLED detract from the overall clarity of the primary methods and results. We have removed this analysis from the manuscript.

**- Relevance of this particular analysis to IHL arguments and what further work needs to be done to make these links more robust.**

Thank you. We have expanded on the relevance of this analysis to IHL arguments in the Discussion section (page 16, lines 472-479):

*“This study identified attacks on medical facilities occurring when Mariupol was under siege by Russia.^54^ The Armed Forces of the Russian Federation have been identified as the actor associated with aerial strikes that would be expected to result in the damage to medical facilities observed in this study.^55^ To our knowledge, there is no evidence that Ukrainian troops conducted airstrikes on any medical facility in Mariupol during the study period. Beyond the broad linkage of damage, two years of research by human rights organizations has identified the specific units of the Armed Forces of the Russian Federation responsible for the extensive destruction of Mariupol.^55^”*

And pages 16-17, lines 480-545:

*“Medical facility locations are increasingly maintained in online databases with information such as building latitude and longitude, footprint polygons, and imagery of signage.^56,57^ The locations of all medical facilities included in this study are available to the public, including to the Armed Forces of the Russian Federation, in a minimum of two databases. Intentional targeting of medical facilities would clearly violate international humanitarian law and would constitute war crimes. The only exception to this rule is in cases where the forces targeting medical facilities have clear reason to believe that those facilities were used for non-medical military purposes. One such claim has been found baseless.^55^ Even if medical facilities were used for military purposes, the prohibition on causing excessive harm to civilians and civilian objects would in most cases prohibit strikes. Moreover, the requirement that armed forces take precautions to avoid damaging medical facilities means that the extensive damage to medical facilities in Mariupol would violate international humanitarian law regardless of whether there was a specific intent to target medical facilities.^58^”*

We have also highlighted an additional area for future research (Discussion section, page 19, lines 637-640):

*“Future satellite imagery analysis of aerial spray may also be used to detect the source of attacks, which represents an important contribution to attribution. Finally, future research may include developing real-time remote indictors of medical facility functioning to serve operations in ongoing conflict.”*

**And reviewer 1 on:**
**- Distinguishing between conflict events and direct attacks**

We thank the Editor for highlighting this important point. International humanitarian law prohibits damage to medical facilities. As such, any damage to a medical facility is widely defined as an attack on health. We have added this justification to our manuscript in the Introduction and Methods sections.

Introduction section, page 3, lines 49-51:

*“Serious violations of these protections, including “extensive destruction and appropriation of property” that is protected, may constitute war crimes.^1–3^”*

Methods section, page 9, lines 244-245:

*“Damage to medical facilities is an observable form of violence against medical facilities.^10^”*

**- Attribution of damage to Ukrainian and Russian Forces respectively
- More detail on methods for joining ACCLED and satellite data**

We thank the Editor for highlighting the lack of clarity around the use of the ACLED data. ACLED event latitude/longitude point data were linked to building polygons in a spatial join. Nonetheless, due to the limitations identified by the Editor and Reviewer, noted above, we have removed this component of the analysis.

**- Tempering expectations of attribution links between damage and perpetrator based on your findings, and what future research can be done to improve this.**

We thank the Editor for raising this point. We have removed the tangential attribution analysis of ACLED data from the manuscript. Instead, we have provided sources attributing the aerial strikes, which would cause the external damage observed by satellite imagery, to Russian Forces. We have also noted there is no evidence, to our knowledge, that Ukraine dropped bombs on health facilities in Mariupol. We have added this information to the Discussion section (Discussion section, page 16, lines 457-463):

*“The Armed Forces of the Russian Federation have been identified as the actor associated with aerial strikes that would be expected to result in the damage to medical facilities observed in this study.^55^ To our knowledge, there is no evidence that Ukrainian troops conducted airstrikes on any medical facility in Mariupol during the study period. Beyond the broad linkage of damage, two years of research by human rights organizations has identified the specific units of the Armed Forces of the Russian Federation responsible for the extensive destruction of Mariupol.^55^”*

**Though this may seem obvious, please also explain in the introduction how physical damage to health facilities likely results in reduced capacity, poor access, fear for health workers to come to work, etc.**

We thank the Editor for this comment and to engage with the literature on the impacts of the conflict on health. We have added a description of the impacts of damage to health facilities to the Introduction section (page 3, lines 58-61):

*“Beyond direct harm to health care workers and patients, attacks on health care in Ukraine have severely disrupted access to health care due to security concerns, restricted mobility, broken supply chains, and high rates of absenteeism and health workforce turnover^17,18^”*

**I am also interested in the lack of involvement of Ukrainian co-investigators from Mariupal, and the potential insights into the methods and results which may have been missed. Did the team attempt to gain local knowledge, and are there Ukranian authors left out for reasons of their own safety?**

We thank the Editor for this point. Due to the ongoing conflict, for their own safety and operational capacity, our Ukrainian partners opted not to be publicly recognized. We have; however, acknowledged their participation in the Acknowledgements section (page 29, line 800):

*“The authors are grateful for the contributions of anonymous Ukrainian colleagues.”*

**Feel free to mention where you disagree with the comments or feel that you have a better suggestion than what is offered.**

**Reviewer #1:**

**It is notable and unfortunate that no colleagues from Ukrainian institutions were included as authors in this research, especially given how local insight could have enriched interpretation of findings.**

We thank the Reviewer for their attention to this matter. Due to the ongoing conflict and potential for disruption to their operational capacity, our Ukrainian partners opted not to be publicly included as authors. However, we have acknowledged their participation in the Acknowledgements section (page 29, line 800):

*“The authors are grateful for the contributions of anonymous Ukrainian colleagues.”*

**While the paper outlines a quality, reproducible method for assessing damage to health infrastructure during conflict, it seems to overstate its significance in building evidence to hold perpetrators accountable. Documenting damage to health facilities as a result of general conflict without strong causal/attribution links is distant from providing sufficient evidence to hold perpetrators accountable for IHL violations. This is especially true when both sides of the conflict are listed as the main actors for conflict events causing damage to health facilities.**

We thank the Reviewer for raising this point. We agree that the attribution arguments made based on the linkage with ACLED data, without temporal alignment, are weak. It is clear from the reviewer reports that this linkage has detracted from the overall goal of the manuscript, and has thus been removed.

The overall goal of this study is to quantify the effect of conflict on medical infrastructure using reproducible methods. We have removed the tangential attribution analysis of ACLED data from the manuscript. Instead, we have provided sources attributing the aerial strikes, which would cause the external damage observed by satellite imagery, to Russian Forces. We have also noted there is no evidence, to our knowledge, that Ukraine dropped bombs on health facilities in Mariupol. We have added this information to the Discussion section (Discussion section, page 16, lines 457-463):

*“The Armed Forces of the Russian Federation have been identified as the actor associated with aerial strikes that would be expected to result in the damage to medical facilities observed in this study.^55^ To our knowledge, there is no evidence that Ukrainian troops conducted airstrikes on any medical facility in Mariupol during the study period. Beyond the broad linkage of damage, two years of research by human rights organizations has identified the specific units of the Armed Forces of the Russian Federation responsible for the extensive destruction of Mariupol.^55^”*

**There needs to be a clear distinction between the following concepts and appropriate application throughout the manuscript, as both are conflated in the paper:

General conflict events which results in collateral damage to health facilities vs. direct attacks on health facilities. As insufficient detail is provided in the methods section on how attacks are matched with facilities, it is difficult to know which is addressed. Consistent language should be used on whether they are conflict events or direct attacks.**

**Documenting attacks on health facilities vs. estimating impact of conflict events/attacks on health facilities**

We thank the Reviewer for this opportunity for clarification. International humanitarian law prohibits damage to medical facilities. We have clarified the purpose of our study, which is to assess the extent of damage to medical facilities during the siege in the Introduction and Methods sections.

Introduction section, page 3, lines 49-51:

*“Serious violations of these protections, including “extensive destruction and appropriation of property” that is protected, may constitute war crimes.^1–3^”*

Methods section, page 9, lines 244-245:

*“Damage to medical facilities is an observable form of violence against medical facilities.^10^”*

**Major bias introduced by omitting any descriptive analysis or comments on the fact that 18 of the 56 (32%) damaged facilities list the Ukrainian military as the main actor. The paper implies that all attacks and damage are the result of Russian Federation attacks.**

We have removed the ACLED analysis from this manuscript. In this case, we are unaware of any evidence that the Ukrainian military was bombing Ukrainian hospitals. We hypothesize that the observed structural bombardment damage was a result of the siege. While beyond the scope of this study, we suggest future attribution investigations can advance these results in the Discussion section (page 20, lines 621-624):

*“Future satellite imagery analysis of aerial spray may also be used to detect the source of attacks, which represents an important contribution to attribution.”*

**This paper overlooks key literature on the topic, including:
o** [**https://phr.org/our-work/resources/russias-assault-on-ukraines-health-care-system/**](https://phr.org/our-work/resources/russias-assault-on-ukraines-health-care-system/) **o** [**https://conflictandhealth.biomedcentral.com/articles/10.1186/s13031-023-00557-2**](https://conflictandhealth.biomedcentral.com/articles/10.1186/s13031-023-00557-2) **o and does not give appropriate attention to a third (though it’s referenced in 32)** [**https://drive.google.com/file/d/1tJyyae_9eBF_IfkPiDb5i9D2Bqb-**](https://drive.google.com/file/d/1tJyyae_9eBF_IfkPiDb5i9D2Bqb-) **2n5w/view .**

**This paper seems to be a secondary approach to UHC’s 2022 report on the same topic. As such, this should be referenced more clearly and prominently in the background or discussion.**

We thank the Reviewer for highlighting this relevant literature for inclusion. We have engaged with the suggested literature, and more, published since our first drafting of this manuscript (<https://www.medrxiv.org/content/10.1101/2023.08.01.23293508v1>). We have also clarified that the unique contribution of this independent approach involves sole reliance on a “definitive” source and the validation of health facility locations. We have also reported alignment of our results with those of UHC in the Discussion section (page 15, lines 414-420):

*“Our results are also aligned with those of the UHC, which reported damage or destruction to 77% of facilities in Mariupol.^27^ The similar proportions of observed damage identified by satellite imagery, considered “definitive,”^34^ and evidence drawing from open data sources suggests the validity of the approach used in this investigation. Furthermore, satellite imagery damage assessment of cross-referenced facilities may represent an opportunity to standardize reporting for direct comparisons within and across conflicts.”*

**Methods, results, and discussion should be strengthened.
o While the background focuses on weaknesses in documenting attacks on health facilities, a weak methods section makes it unclear whether this paper provides an approach that improves upon existing surveillance activities.
o Detail is needed on cleaning of the ACLED dataset and how facilities and conflict events are matched. Without this, it is difficult to ascertain the likelihood that the damage is attributable to a specific conflict event/actor.
o Results section doesn’t provide descriptive summaries of conflict events and cherry picks certain messages (i.e. highlighting Russian attacks without acknowledging Ukrainian attacks matched with damaged facilities)**

We appreciate the Reviewer’s concerns regarding the ACLED data and agree with the limitations in using this data to suggest attribution. Of note, the ACLED event does not link the actor to the event type, thus identifying both the perpetrators conducting an aerial strike as well as affected actors. We have thus removed this part of our analysis. We have strengthened the Methods section. A few additions are noted below, with more in response to the following specific comments.

Page 6, lines 155-156:

*“This observational, pre-post quasi-experimental study identifies the effects of Russia’s invasion on external damage to medical facilities within Mariupol during the seige.”*

Page 7, lines 194-195:

*“The included databases are generated independently of each other.”*

Page 9, lines 235-236:

*“Building footprint and height were derived from OpenStreetMap.^45^ For facilities comprised of multiple buildings, polygons were grouped into a complex.”*

**o Discussion section does not explore or address existing literature on the topic or possible alternative explanations for findings. While understandably this paper can’t make the link between damage and the level of evidence for an IHL conviction, the paper should be framed as a building block and provide feedback on how future research can build on this step to move towards holding perpetrators accountable. Currently, it reads as though the paper on its own will lead towards more perpetrator accountability.**

We thank the Reviewer for highlighting additional literature to explore. We have engaged with the additional following sources in the Discussion section:

*Kim, H.-J. et al. Typology and implications of verified attacks on health care in Ukraine in the first 18 months of war. PLOS Glob. Public Health* ***4****, e0003064 (2024).*

*Barten, D. G. et al. Attacks on Ukrainian healthcare facilities during the first year of the full-scale Russian invasion of Ukraine. Confl. Health* ***17****, 57 (2023).*

*Ukrainian Healthcare Center. Destruction of Healthcare in Mariupol. https://uhc.org.ua/en/2023/01/04/healthcare-in-mariupol/ (2022).*

*Cox, K., Flint, R., Favaro, M., Slapakova, L. & Harris, R. Researching Violence against Healthcare: Gaps and Priorities. https://www.elrha.org/wp-content/themes/elrha/pdf/elrha-and-icrc-violence-against-health-care-full-report-010720-digital.pdf (2020).*

*Surveillance System for Attacks on Health Care. https://extranet.who.int/ssa/Index.aspx (2023).*

*Human Rights Watch. ‘Our City Was Gone’ Russia’s Devastation of Mariupol, Ukraine. https://www.hrw.org/sites/default/files/media_2024/02/ukraine0224web.pdf (2024).*

We have clarified the contribution of this analysis, which is demonstrating the feasibility of assessing the extent of damage to medical complexes using cross-referenced location data (Discussion section, pages 15, lines 402-408) accounting for facility size:

*“We present independent evidence of widespread damage to cross-referenced medical facilities during the besiegement of Mariupol. This study also demonstrates the feasibility of generating reproducible and replicable^51^ estimates of the effects of conflict on medical facilities using commercially and publicly available data sources, a previously noted gap in the literature analyzing attacks on healthcare.^52^ These methods represent an opportunity to strengthen existing surveillance systems of attacks on medical facilities.”*

**Referencing:
o Many references are not accurately represented or statements in the manuscript are not backed up by the provided references. (Examples provided below)
o Many references are missing links, which are especially important for grey literature. Ex. Reference 32 does not produce any immediate results in a Google search with that exact title.**

We thank the Reviewer for noting this issue and have ensured the grey literature references include links.

**Data sources - Health facility list
o Was any effort made to confirm the functionality of the health facilities prior to the conflict events? Evidence from other settings suggests there is often a large exodus of health care workforce when conflict begins.**

We thank the Reviewer for raising this point. Evaluating health facility functioning was outside the scope of this study. Notably, medical facilities remain protected under international humanitarian law even if not currently functioning. Our Ukrainian partners were, nonetheless, interested in damage status as a proxy for functionality. Future research could explore remote indicators of functionality to improve the operational relevance of this type of analysis. We have suggested this in the Discussion section (page 20, lines 623-624):

*“Finally, future research may include developing real-time remote indictors of medical facility functioning to serve operations in ongoing conflict.”*

**o Was any effort made to confirm the accuracy of medical facility location data? Publicly available data on this is known to be wildly incomplete/inaccurate in other conflict settings.**

*We thank the Review for this query. The extensive cross-referencing we performed to confirm the accuracy of medical facility locations is a strength of this paper, which, to our knowledge, had not been done in similar studies.*

**o Doesn’t clearly define a health facility for the purposes of this study or discuss how different datasets might classify facilities differently. For example, UHC considers the 12 separate outpatient points in 8 communities that make up the Mariupol PHC as separate facilities. Does OSM do the same?**

We appreciate the Reviewer’s query about facility type (e.g., clinic, hospital). Information about facility type was inconsistently available across databases. The search strategy used for identifying and cross-referencing medical facilities is presented in the Methods section (pages 7-8, lines 211-213).

**o Would be helpful to state how each health facility dataset sources its data to ensure Wikimapia does not source data from OSM/Google for example. This would lead to invalid verification by multiple sources.**

We thank the Reviewer for raising this point. The included datasets were purposefully chosen as they do not source each other. We have added this clarification to the Methods section (page 7, line 194-195):

*“The included databases are generated independent of each other.”*

**Specific feedback:

The title should be made more specific, as its current phrasing implies a more comprehensive exploration of effects, while this study only examines external damage to medical facility physical infrastructure during the conflict period.**

We thank the Reviewer for this suggestion. We have updated the title as follows:

*“The effect of conflict on damage to medical facilities in Mariupol, Ukraine: a quasi-experimental study”*

**The validity of the following statement is questionable: “This is the first geographically comprehensive pre-post study of the effects of an ongoing conflict on specially protected medical infrastructure.” (lines 35-36). It is unclear how this is different from previously published studies other than using remote sensing data:** [**https://conflictandhealth.biomedcentral.com/articles/10.1186/s13031-023-00557-2**](https://conflictandhealth.biomedcentral.com/articles/10.1186/s13031-023-00557-2) **. And geographically comprehensive to Mariupol?**

We thank the Reviewer for the opportunity to clarify the novelty of this analysis, which is three-fold: This is the first study to: 1) cross-reference health facility location data, 2) conduct replicable reproducible damage assessments of fully publicly and commercially available data, including a baseline assessment, and 3) test the expected association between building size and sustained damage. We have updated the line in question as follows (Abstract, page 2, lines 34-36):

*“This is the first cross-referenced pre-post census study of the effects of an ongoing conflict on specially protected medical infrastructure.”*

**Line 47 – Need to be clear on whether this clause refers to Ukraine or globally. If globally, which it seems to be, the provided reference does not apply. There are more appropriate references, including the SHCC report.**

*We thank the Reviewer for this comment and for pointing us toward the suggested reference. We have updated the reference to the SHCC report.*

**Lines 48-56 – PHR has done one of the more robust efforts to document attacks on health care in Syria and Ukraine, which should be included here, especially as you reference several papers pertaining to Syria in the background.**

Thank you for this suggestion. We have added references to the impressive PHR literature relevant to Ukraine and Syria:

*The Syrian Conflict: Eight Years of Devastation and Destruction of the Health System. PHR* [*https://phr.org/our-work/resources/the-syrian-conflict-eight-years-of-devastation-and-destruction-of-the-health-system/*](https://phr.org/our-work/resources/the-syrian-conflict-eight-years-of-devastation-and-destruction-of-the-health-system/)*.*

*Physicians for Human Rights, Ukrainian Healthcare Center, eyeWitness to Atrocities, Media Initiative for Human Rights & Insecurity Insight. Destruction and Devastation: One Year of Russia’s Assault on Ukraine’s Health Care System. https://phr.org/our-work/resources/russias-assault-on-ukraines-health-care-system/ (2023).*

**Line 58-60 – This statement is misleading. The topic sentence focuses on attacks on medical facilities, but the two papers referenced address grave violations against children (not including attacks on health) and attacks on education. The following sentences then jump back to statements related to attacks on health. This should be rewritten for clarity and accuracy.**

We thank the Reviewer for highlighting this issue. We have removed this sentence as we find that the different focus, even rewritten, could be confusing and detract from the overall argument.

**Lines 57-68 – Conflation between documenting attacks and estimating the effects of attacks on health. This should be clearly separated.**

We appreciate the opportunity to edit this section as suggested by the Reviewer. We have revised the section as follows (Introduction section, pages 4-5, lines 76-109):

*“Despite the importance of documenting attacks on medical facilities,^8^ coordinated and systematic data collection efforts have lagged. Evidence of patterns of attacks on healthcare reported in the peer-reviewed literature is weak, with only 11% of studies involving the collection of quantitative data.^8^ Existing public surveillance data is also of insufficient quantity and quality.^10^ Although there are reasonable limitations to the responsible sharing of location information in active conflict settings, databases lacking geocoding sufficient for subnational disaggregation preclude independent verification.^10^ Analyses of georeferenced databases often fail to corroborate health facility location information, and reliance on such datasets may produce erroneous results.^28,29^ Physical building attributes relevant for understanding probabilistic patterns of attacks, such as building footprints, have, to our knowledge, not been assessed in investigations of damage to medical facilities. Finally, the absence of standardized incident classification and verification across data sources and privileging of particular conflicts and forms of attacks further limit validity.^30,31^ These weaknesses have produced a restricted, non-representative evidence base^32^ with a particular dearth of quantitative data. The absence of systematic surveillance of attacks on protected medical facilities during conflict makes it difficult to identify trends,^33^ weakens efforts to prevent future attacks,^21,30^ and limits the potential of accountability mechanisms.”*

**Line 66 - Has grammatical errors and doesn’t make sense.**

We thank the Reviewer for the opportunity to correct the grammatical errors. We have revised this sentence as follows:

“*Finally, the absence of standardized incident classification and verification across data sources and privileging of particular conflicts and forms of attacks further limit validity.^30,31^*

**Line 110-111 – This should be made more specific (along with the title) to specify that this study is on the effects of conflict on external medical facility physical infrastructure.**

We thank the Reviewer for raising this point. We have updated both this sentence:

*“This observational, pre-post quasi-experimental study identifies the effects of Russia’s invasion on external damage to medical facilities within Mariupol during the seige.”*

As well as the title:

*“The effect of conflict on damage to medical facilities in Mariupol, Ukraine: a quasi-experimental study”*

**Lines 129-131 – Missing references - “Of note, past examples of geolocated medical facilities have relied on a single data source, although such databases are known to have heterogenous content and quality.”**

We thank the Reviewer for this comment. One of the major strengths of this study is the cross-referencing of medical facility location geocoordinates. We have added the following references describing data heterogeneity and possible implications for analysis:

*Weiss, D. J. et al. Global maps of travel time to healthcare facilities. Nat. Med.* ***26****, 1835–1838 (2020).*

*Jacquez, G. M. A research agenda: Does geocoding positional error matter in health GIS studies? Spat. Spatio-Temporal Epidemiol.* ***3****, 7–16 (2012).*

**Lines 131-132 – Increases likelihood for improved validity of findings. Cross-referencing bad data, doesn’t lead to more valid findings.**

We thank the Reviewer for raising this point. We do not consider any of the included datasets bad, and in fact, several have been incorporated into existing analyses and publications independent of external validation. We do anticipate error in georeferenced databases and aimed to minimize this potential in our analysis by cross-referencing independent sources. Additionally, the georeferenced health facility locations used in this analysis were available to military actors, who thus could have been expected to apply the principles of protection, precaution, and proportionality to these locations.

**Lines 154-160 – Is there more than one polygon for each facility? Why?**

We thank the Reviewer for this opportunity to clarify. Yes, in some cases, for example, a large hospital, one facility may be comprised of multiple buildings, and thus multiple polygons. In these cases, complex area, maximum height, and volume were calculated for the entire structure. We have updated the Methods section as follows (Methods section, page 9, lines 235-236):

*“For facilities comprised of multiple buildings, polygons were grouped into a complex.”*

**Line 164 – What was the result of the baseline structural assessment? Were facilities classified as damaged dropped?**

We thank the Reviewer for this opportunity to provide clarification. No facilities were classified as damaged at baseline (Results section, page 13, lines 356-357):

*“All 73 medical facilities included in this study were confirmed intact at baseline.”*

**Line 196 – Narrative references IWG-SEM was used, but table references an adapted version of IWG-ES. Why?**

We thank the Reviewer for identifying this typo. We have corrected “IWG-ES” to “IWG-SEM” in the table description (Methods section, page 13, line 284).

**Lines 200-202 - Unclear why a distinction wasn’t made between minor and major damage, as one could still calculate the proportion of facilities damaged from minor/major classifications. Especially as the paper is framed around documenting IHL violations to hold actors accountable, being able to categorize whether the damage is superficial or affects health service functionality seems very important.**

We thank the Reviewer for raising this important point. Distinction between IWG-SEM damage classification levels (i.e., minor vs. major) was not consistently possible due to imagery variation. Thus, we adopted a binary approach. The overall goal of this study was to assess the pattern of damage to medical complexes during the besiegement. We argue that structural damage in and of itself represents and is consistent with the IHL definitions of potential war crimes (Introduction section, page 3, lines 47-51):

*“These protections are enshrined in the 1949 Geneva Conventions^1–3^ and the two additional protocols to the Conventions.^4,5^ Serious violations of these protections, including “extensive destruction and appropriation of property” that is protected, may constitute war crimes.^1–3^”*

**Lines 202-214 – In the binary system this was classified as not damaged due to insufficient image quality? It is unclear from the example how you distinguish between minor damage and indeterminate damage which would land in different binary classifications.**

We thank the Reviewer for this opportunity to clarify our methods. Figure 1 presents an example of indeterminate damage severity. The building would be classified as damaged, as there are clear signs of damage. We have updated the Figure title and caption as follows (Methods section, pages 12, lines 296-300):

*“****Figure 1. Damage of indeterminate severity.*** *Discoloration along the western portion of the roof and clear signs of damage in the center of the roof are visible. However, the severity of the damage, assessed as the structural integrity of the roof, is indeterminate. Due to the clear signs of damage, this structure would be classified as “damaged” in the adapted IWG-SEM schema used in this study.”*

**Lines 222-230 – This leaves a lot to be desired in terms of whether the ACLED data was cleaned and the parameters of the joins. More is needed on how the ACLED data was matched to medical facility polygons. Was the dataset filtered based on location accuracy scores in the ACLED dataset? Were event types filtered out, such as riots/civil unrest, where a mechanism for damage from military attacks would not be present? Was there a cutoff for maximum distance of a conflict event to a health facility? Date range? etc.**

We thank the Reviewer for their comments regarding the ACLED dataset. Initially, we matched the exact point coordinates of ACLED events to building polygons. Ultimately, we have removed this dataset from the presented analysis due to underlying issues with the presentation of the ACLED data related to attribution, which was the main motivator for its inclusion.

**Line 250 – Was any effort made to confirm the functionality of the health facilities at the start of the conflict?**

We thank the Reviewer for raising this point. Evaluating health facility functioning was outside the scope of this study. Our Ukrainian partners were, nonetheless, interested in damage status as a proxy for functionality. Future research could explore remote indicators of functionality to improve the operational relevance of this type of analysis. We have suggested this in the Discussion section (page 20, lines 623-624):

*“Finally, future research may include developing real-time remote indictors of medical facility functioning to serve operations in ongoing conflict.”*

**Lines 274-277 – Is there a reference to support this bold statement? There seem to be other possible explanations. The spread of facility sizes is not that large, so is there evidence to support the assumption that larger facilities would be more likely to be hit? It seems more likely if comparing facilities 100sqm to 3000sqm, but actual facility footprints don’t seem to vary dramatically. There are presumably more smaller facilities, so stratification would be helpful to know whether the n for large facilities is sufficient to compare. Is there evidence from other studies to suggest that one would expect there to be variation in damage sustained based on these modest size differences? Lots of other explanations seem possible…proximity to strategic targets, large proportion of the area conflict affected, etc.**

We thank the Reviewer for the opportunity to expand this presentation of results. This statement is supported by the absence of statistical association between building size and damage status, as presented in Table 3. We have added an interpretation of the variance, or range in building size to this point (Results section, pages 14, lines 379-381):

*“Variation in facility size was notable: the facility with the largest footprint was 2.4-fold larger than the facility with the smallest footprint. The volume of the largest facility was 4.4-fold greater relative to the facility with the least volume.”*

**Lines 280-283 – these numbers don’t sum to 46. Also, more description on what matched means (should be clarified in the methods) is essential to understand the implications of being matched with a conflict event.**

We thank the Reviewer for their attention to this detail. ACLED reports multiple conflict types per event, although not linked to perpetrator. We have removed the linkage to ACLED data.

**Lines 283-286 – Would be helpful to see a better descriptive summary of the characteristics of matched conflict events. What proportion of conflict events were attributed to the Russian Federation? If not the Russian military, then which actor was identified? What was the breakdown by conflict event type? If the Ukrainian military is responsible for one third of the conflict events matched to damaged health facilities, how does this influence the results, discussion, and conclusions?**

We thank the Reviewer for their thoughtful comments regarding the linkage to ACLED data. In response, we have removed the linkage to ACLED data, described in detail above.

**Lines 292-293 – This statement, like the title of the paper, should be tempered. While the effects of conflict on health facilities can be wide ranging (staffing, service availability, service use, facility functionality, supply chain, etc.), this paper is only able to identify whether any external damage to a facility occurred, not including severity of damage which might further implicate functionality effects. Furthermore, the attribution links between conflict events and damage are weak, especially in the presented framing of IHL accountability.**

We thank the Reviewer for this comment. We have revised the statement as follows (Discussion section, pages 15, lines 402-408):

*“We present independent evidence of widespread damage to cross-referenced medical facilities during the besiegement of Mariupol. This study also demonstrates the feasibility of generating reproducible and replicable^51^ estimates of the effects of conflict on medical facilities using commercially and publicly available data sources, a previously noted gap in the literature analyzing attacks on healthcare.^52^ These methods represent an opportunity to strengthen existing surveillance systems of attacks on medical facilities.”*

**Line 299 – Typo in reference. Also, the sentence indicates the referenced sources cannot be compared due to methodological differences, but reference 43 simply pulls a number from a news article quoting a politician. There are no methods or data presented behind this statement.**

We thank the Reviewer for noting this typo, which we have corrected.

We have taken the opportunity to revise this statement as well, including noting alignment of our independent, cross-referenced results with existing reports of damaged medical infrastructure (Discussion section, page 15, lines 412-420):

*“The percentage of damage identified in this study is consistent with previous reports that 80% of the health care infrastructure in Mariupol has been destroyed.^27^ Our results are also aligned with those of the UHC, which reported damage or destruction to 77% of facilities in Mariupol.^27^ The similar proportions of observed damage identified by satellite imagery, considered “definitive,”^34^ and evidence drawing from open data sources suggests the validity of the approach used in this investigation. Furthermore, satellite imagery damage assessment of cross-referenced facilities may represent an opportunity to standardize reporting for direct comparisons within and across conflicts.”*

**Lines 297-299 – More unpacking of the differences between the studies would be helpful in understanding how this approach is an improvement on the previous estimates and the implications for future research.**

We thank the Reviewer for the opportunity to expand on the strengths of this study. The evidence presented here represents, for the first time, a cross-referenced “definitive” assessment of medical facilities during an ongoing conflict, accounting for building features relevant to targeting. We have updated this section as described above (Discussion section, pages 17, lines 532-535):

*“A major strength of our study is the pre-post quasi-experimental study design used to estimate the effects of Russia’s siege on medical facility infrastructure by applying reproducible and replicable methods to publicly available and cross-referenced data sources, accounting for building features relevant to targeting.”*

**Lines 303-305 – Again, would be helpful to have a descriptive summary of the actors and weaponry associated with the included attacks somewhere in the paper.**

*We have removed the ACLED data from this analysis due to variable quality and a lack of linkage between multiple actors and events in the ACLED database.*

**Lines 306-307 – This statement seems highly susceptible to reporting bias, i.e. heavy weaponry and damage at large facilities would be more likely to be detected and reported than small arms attacks on small facilities. This also cites a system that was highlighted as insufficient in the background section. These, and other considerations, should be noted.**

We thank the Reviewer for this comment. We agree that small arms attacks may be less likely to be reported. Nonetheless, we have positioned our findings in the context of the existing literature. We have reported that this work only documents damage visible from space in the Limitations section (page 17, lines 543-545):

*“First, damage assessments conducted primarily using satellite imagery are limited to visible features and may not always show damage to a structure’s sides and interior.”*

**Line 329 – Would the study have been possible without the commercial data from the State Department?**

This study benefitted from financial support, as described in the Financial Disclosure. Maxar operates a humanitarian platform in which imagery is distributed free of charge upon request.

**Lines 327-331 – This statement seems far too strong for the evidence presented in the paper. Very weak attribution links are presented and this does not address any of the major hurdles to holding IHL perpetrators accountable.**

We thank the Reviewer for this comment. Indeed, we believe this is the first census of damage to health facilities in Mariupol, Ukraine using publically and commercially, cross-referenced databases. The results presented in this paper may be considered alongside other evidence, including that pertaining to perpetration (Discussion section, page 16, lines 457-463):

*“The Armed Forces of the Russian Federation have been identified as the actor associated with aerial strikes that would be expected to result in the damage to medical facilities observed in this study.^55^ To our knowledge, there is no evidence that Ukrainian troops conducted airstrikes on any medical facility in Mariupol during the study period. Beyond the broad linkage of damage, two years of research by human rights organizations has identified the specific units of the Armed Forces of the Russian Federation responsible for the extensive destruction of Mariupol.^55^”*

**Lines 331-335 – Isn’t making the evidence available to all parties requisite in trials?**

We thank the Reviewer for the opportunity to clarify this point. We have revised the text as follows (Discussion section, page 17, lines 537-540):

*“Moreover, the analysis of publicly available data for the generation of evidence is available to all actors and in a variety of settings, from international to domestic operational theaters to courtrooms and diplomatic settings.”*

**Reviewer #2:** 

**General Comments:

I appreciated the opportunity to review this submission.

The paper presents itself as a quasi-experimental study of pre- and post- invasion analysis of attacks on Mariupol, Ukraine medical facilities, measured as building damage, during the three month siege by Russian forces in 2022. They derive medical facilities from multiple cross-referenced open-sourced and government data sets, create building geolocated polygon footprints, and use satellite imagery with routine classification analysis with an accepted methodology to quantify prevalence of damage and discuss these findings within the principles and framework of IHL.

In general, quasi-experimental designs, by definition, help illuminate causation and from the spatial and temporal standpoint, the paper accomplishes that. Where it falls short is attempting to relate events from the ACLED database with damage without presenting any actual time/place analysis of that data, at which point it detracts from the study design and purpose. Please see my comments below for more specifics – I think it can be done in a way that compliments and enforces the paper’s focus.**

We thank the Reviewer for their thoughtful assessment of our work.

**The study also successfully argues for more robust infrastructure damage/medical attack surveillance systems upon which they depend, although that is discussed in the Introduction and is not mentioned in the Discussion. But the point is well-taken.**

We thank the Reviewer for highlighting this point. We have further articulated this in the Discussion section (pages 15, lines 402-408):

*“We present independent evidence of widespread damage to cross-referenced medical facilities during the besiegement of Mariupol. This study also demonstrates the feasibility of generating reproducible and replicable^51^ estimates of the effects of conflict on medical facilities using commercially and publicly available data sources, a previously noted gap in the literature analyzing attacks on healthcare.^52^ These methods represent an opportunity to strengthen existing surveillance systems of attacks on medical facilities.”*

**A few other general comments:

1. The derivation of the facility dataset seems reasonable and thoughtful. In fact, the researchers have done due diligence to minimize data gaps that frequently occur in places with on-going conflict.**

We thank the Reviewer for this comment. We believe confirming the facility type of all buildings included in this analysis through cross-referencing is important for accountability.

**2. Despite the conservative definition of the binary damage classifications (well-explained in the Limitations), the amount of damage was disturbingly large and that fact alone renders this study valuable in raising IHL allegations.**

We agree with the Reviewer’s assessment of the findings – while we adopted a conservative definition of damage, we find that the magnitude of damage raises concerns about the adherence to IHL, and is consistent with other sources using alternative data collection methods.

**3. I appreciate the fact that the indiscriminate pattern of damage is brought out in both the Results and Discussion. The strength of the paper highlights what is often missed – not that Russian forces were targeting medical facilities (they may well have), but their obligation under IHL is to not be indiscriminate and not to be disproportionate when their obligation is protecting civilians and civilian infrastructure. What that in mind, I would suggest also making a proportionality argument given the amount of destruction of medical infrastructure in a short time (demonstrated in the analysis), especially, as you later note, realizing that damage is undercounted and underappreciated from your conservative imagery classification.**

We are grateful to the Reviewer for this suggestion. We have revised the Limitations section to note the requirement of proportionality (page 18, lines 573-576):

*“Yet, even if Russia’s armed forces had military objectives in conducting the attacks, the special protected status of medical facilities should have resulted in the application of the principles of proportionality and precaution.”*

**The authors provide a compelling introduction and rationale defining the need for the study and its role in providing additional evidence in a milieu as fraught as the Mariupol siege.
Nonetheless, the authors should make it clear that this study is still a ‘one off’ point in time analysis that has the potential to add to a larger body of evidence if and, hopefully, when it’s needed for international legal bodies to confer.**

We thank the Reviewer for the opportunity to position our work in the greater portfolio of evidence of attacks on health during this conflict. We have updated the Conclusions as follows (page 20, lines 627-637):

*“To our knowledge, this is the first evidence of widespread damage to cross-referenced facilities in a pre-post quasi-experimental study of the effects of war on urban medical infrastructure. As such, the reproducible methods, incorporating publicly and commercially available data, have the potential to make substantial contributions to the international justice system by providing real-time estimates of damage to specially protected civilian objects. By combining the strengths of satellite imagery analysis in nonpermissive environments with publicly available data sources of medical facility infrastructure, this study demonstrates the power of data amalgamation for creating outputs that were, until recently, inaccessible. The findings presented here support the existing evidentiary basis that attacks on Ukraine’s health care system constitute war crimes.^61^”*

**1. Line 66 – confusing sentence – change perhaps to ‘absence of impact estimations’?**

We thank the Reviewer for the opportunity to revise the sentence, as follows (Introduction section, page 4, lines 87-89):

*“The absence of systematic surveillance of attacks on protected medical facilities during conflict makes it difficult to identify trends,^32^ weakens efforts to prevent future attacks,^20,29^ and limits the potential of accountability mechanisms.”*

**2. Small change: Ref 36 should be “International Working Group” (not Interagency) – please provide the link to public documents, in this case:** [**https://www.un-spider.org/sites/default/files/IWG_SEM_Guidelines_Building%20Damage%20Assessment_v1.0.pdf**](https://www.un-spider.org/sites/default/files/IWG_SEM_Guidelines_Building%20Damage%20Assessment_v1.0.pdf)**.**

We thank the Reviewer for noting this error. We have updated this reference and included the URL.

*“International Working Group on Satellite-based Emergency Mapping. Emergency Mapping Guidelines: Building Damage Assessment. https://www.un-spider.org/sites/default/files/IWG_SEM_Guidelines_Building%20Damage%20Assessment_v1.0.pdf. (2018).”*

**3. Methods –
a. Thanks for introducing us to Natural Earth! Appreciate the need and use for multi-scaled data sets.**

**b. Agree with using multiple facility datasets and cross-referencing them. This adds strength to the paper and mitigates the limitation of ‘completeness’ in an active conflict zone.**

We thank the Reviewer for appreciating the need for independent cross-referencing of geolocated facilities. We have also added the following references describing the importance of cross-referencing geolocated data:

*Weiss, D. J. et al. Global maps of travel time to healthcare facilities. Nat. Med.* ***26****, 1835–1838 (2020).*

*Jacquez, G. M. A research agenda: Does geocoding positional error matter in health GIS studies? Spat. Spatio-Temporal Epidemiol.* ***3****, 7–16 (2012).*

**c. Line 102 – should be ‘km2’**

We thank the Reviewer for highlighting this error - we have corrected the text as follows (Methods section, page 6, lines 147).

**d. Please address any potential types of error in the process of deriving facility polygons.**

We thank the Reviewer for the opportunity to elaborate in the process for creating facility polygons. For several facilities included in this study, multiple building polygons were combined to create a complex polygon (Methods section, page 9, lines 235-236):

*“For facilities comprised of multiple buildings, polygons were grouped into a complex.”*

**e. For clarity – the building polygons consisted of the footprint of the building only and not the land that it was on, yes? Also, when Google Maps and Yandex were used to determine number of floors, was this Street View? If so, address any potential sources of error with this approach.**

We thank the Reviewer for the opportunity to clarify our procedures – yes, the building footprints consisted of the building only. We have updated the Methods section accordingly (page 9, line 235):

*“Building footprint and height were derived from OpenStreetMap.^45^”*

**f. Were the two imagery analysts classifying independently? I assumed so, but please state.**

*Again, we thank the Reviewer for the opportunity to clarify this point. The damage assessments were conducted independently by two imagery analysts. We have updated the text in the Methods section (page 10, lines 259-261):*

*“Multi-temporal change detection between pre- and post-siege imagery was conducted independently by a minimum of two geospatial analysts. Disagreements were resolved via consultation with a third analyst.”*

**g. What was the utility of classifying any imagery with significant cloud cover during the siege (since you have other imagery available without cloud distortion during that time)?**

We thank the Reviewer for noting the opportunity to clarify the methods. We only assessed images with minimal cloud cover (<11%, as described in Table 1).

**h. Analysis – the Clopper-Pearson interval is used appropriately here for the study design of independent binomial outcomes. The authors should state what statistical package they used for calculations.**

We thank the Reviewer for examining the statistical methods presented in this manuscript. Because this is a census study, with no interpolation, we have removed uncertainty around the percentage of damage. All statistical analyses were conducted in Stata version 17.0 (Methods section, page 13, lines 345-346):

*“All analyses were conducted in Stata version 17.0.^49^”*

**4. The authors should consider combining Tables 3 and 4 since they describe the same data outputs.**

We thank the Reviewer for this suggestion. We have consolidated Tables 3 and 4.

**5. The ACLED event information is presented without actual data to demonstrate the connection with building damage. I think you need to provide something here. For instance, when you state the ACLED events were ‘geospatially matched’ (line 280), do you mean they occurred in close proximity to the buildings? While you wisely stop short of directly inferring causality without an analysis (since the ACLED data is not temporally matched), it seems like it would be helpful to clarify what ‘geospatially matched’ actually implies. Also, can the ACLED events be temporally related to the building damage (my fear is that your temporal resolution may not be granular enough)? If you can use a proximity distance measure (event to building) and timestamp of the event to the time of damage, this would be more compelling. The study has shown that considerable damage was done to medical facilities. While it’s probably safe to say that Ukrainian forces were not attacking their own medical facilities during this period, demonstrating that Russian forces inflicting events in time and space during a period of siege would deflect any counter-argument and place the spotlight more squarely on their IHL violations.**

We thank the Reviewer for their insights. By “geospatially matched,” we are referring to directly joining the ACLED event latitude/longitude directly to the building polygons they fall within. However, given limitations of the ACLED data (perpetrators and other actors noted without distinction) and the lack of a temporal linkage, we have dropped the ACLED analysis from this manuscript.

**6. Conclusions. There have been recent studies in the literature using remote sensed imagery on medical facility infrastructure already (Gaza, for example), so the point here should be rephrased to explain how the use of remotely sensed imagery has value in this particular scenario (urban, siege, time-dependent) and how its use is adding validity to the IHL arguments.**

We thank the Reviewer for this suggestion. We have amended the Conclusions as follows (page 20, lines 627-629):

*“To our knowledge, this is the first evidence of widespread damage to cross-referenced facilities in a pre-post quasi-experimental study of the effects of war on urban medical infrastructure.”*

And, page 20, lines 635-637):

*“The findings presented here support the existing evidentiary basis that attacks on Ukraine’s health care system constitute war crimes.^61^”*
